# Five Things to Know About Obesity in Kidney Transplant Candidates

**DOI:** 10.1177/20543581261434826

**Published:** 2026-04-06

**Authors:** Somaya Zahran, Jason Y. Lee, Sunita K. S. Singh

**Affiliations:** 1 Division of Nephrology and Hypertension, Mayo Clinic, Rochester, MN, USA; 2 Division of Urology, Department of Surgery, University Health Network, Toronto, ON, Canada; 3 Ajmera Transplant Centre, University Health Network, Toronto, ON, Canada; 4 Division of Nephrology, Department of Medicine, University Health Network, University of Toronto, ON, Canada

**Keywords:** obesity, kidney transplantation, waiting list, bariatric surgery

## 1. The Burden of Obesity in Kidney Transplant Candidates is Increasing

Obesity prevalence among patients with kidney failure has increased over time, exceeding rates in the general population.^1^ In the United States, nearly 40% of patients with kidney failure and more than 45% of patients on the transplant waiting list have a body mass index (BMI) >30 kg/m².^2,3^ Similar trends have been observed amongst kidney transplant candidates in Canada.^4^ The impact of obesity is associated with decreased access to transplantation.^5^

## 2. Kidney Transplantation Remains the Treatment of Choice in Obese Candidates, but There Are Some Risks Associated With Obesity

While obesity is associated with an increased risk of both short- and long-term complications after transplant, it has been shown that kidney transplantation offers a survival advantage for obese patients across all BMI categories, as compared to remaining on dialysis.^6^ However, in comparison with kidney transplant recipients (KTRs) without obesity, those with a BMI >30 kg/m^2^ are at increased risk of perioperative complications such as death, delayed graft function, acute rejection, longer surgical time, re-operation, surgical site infections, wound dehiscence, incisional hernias, as well as a greater length of stay.^7-11^ Over the long term, obese transplant recipients are more likely to develop posttransplant diabetes mellitus, cardiovascular disease, and death-censored graft loss.^11,12^ Furthermore, among obese recipient candidates, the presence of donor obesity may be associated with an increased risk of death-censored and all-cause graft loss as compared nonobese donors.^13^ Despite these risks, kidney transplantation remains the preferred treatment for most obese transplant candidates.

## 3. Current Clinical Practice Guidelines Do Not Specify a BMI Cutoff for Transplant Eligibility

Existing guidelines recognize that obesity, while associated with increased perioperative risk, should not be an absolute contraindication to kidney transplantation, and that the benefits of transplantation should be weighed against the risks to the obese candidate. The Kidney Disease: Improving Global Outcomes (KDIGO) 2020 guidelines do not specify a BMI cutoff, but rather encourages each transplant program to consider their own resources and skills in caring for obese candidates. KDIGO recommends that all candidates undergo evaluation by a transplant surgeon and suggest offering weight loss interventions, including consideration of bariatric surgery, for those with BMI ≥35 kg/m².

The European Renal Association (ERA) 2022 guidelines^14^ recommend accepting candidates with a BMI of 30–34 kg/m² and encourages individualized evaluation for those with BMI ≥35 kg/m². Bariatric surgery is supported for candidates and recipients with BMI ≥40 kg/m², or ≥35 kg/m² with comorbidities, with laparoscopic sleeve gastrectomy preferred over Roux-en-Y gastric bypass to avoid potential malabsorption of immunosuppressive drugs. Both ERA and KDIGO emphasize shared decision-making, multidisciplinary weight management, and thorough risk counseling.

The Canadian Society of Transplantation 2005 consensus guidelines support supervised weight loss therapy to a target BMI <30 kg/m² although the specific interventions recommended are not explicitly discussed.^15^

In summary, while there is no BMI threshold for kidney transplantation, guidelines are consistent in recognizing that weight loss strategies should be offered to obese transplant candidates.

## 4. The Role of GLP1RA and Bariatric Surgery in Pretransplant Weight Loss Is Evolving

For obese kidney transplant candidates, weight loss before transplantation can improve transplant eligibility and potentially reduce perioperative risks. With the advent of incretin-based therapies, it is likely that recommendations regarding weight loss interventions in the obese transplant candidate will evolve with time. Currently, data on Glucagon-Like Peptide-1 Receptor Agonists (GLP1RA) use are limited as most clinical trials did not enroll patients with kidney failure. Small case series and prospective studies have demonstrated that semaglutide can lead to significant weight loss to achieve kidney transplant eligibility among ineligible obese candidates.^16,17^ The OK-TRANSPLANT 2 vanguard trial is evaluating the use of semaglutide and virtual weight management support versus usual care in achieving weight loss in obese kidney transplant candidates.^18^

Although there are currently no head-to-head studies comparing GLP1RA and bariatric surgery in obese kidney transplant candidates, an economic analysis in obese transplant candidates using Markov modeling found that bariatric surgery was more cost effective than GLP1RA.^19^ Bariatric surgery has been shown to be associated with increased access to transplantation,^20,21^ although access to bariatric surgery varies significantly across provinces, and waiting times can be quite long, often exceeding 12 months.^22,23^

A general comparison of incretin-based therapies versus bariatric surgery is shown in Table 1. More data are needed to better understand how bariatric surgery and/or pharmacological therapy should be implemented for weight loss in the obese transplant candidate.

**Table 1.** Comparison of Incretin-Based Therapies Versus Bariatric Surgery for Weight Loss.

Parameter	GLP1RA or Dual GLP1/GIP RA	Bariatric surgery
% weight loss	15%–21%	30%–80%
Weight regain risk	High rate (>50%) of weight regain with drug discontinuation	Low rate (<10%) of weight regain postsurgery
Coverage and cost	Variable coverage based on insurance plan and indication High cost due to need for lifelong therapy required	Typically covered by provincial health plan One time cost
Side effects	GI side effects (nausea, vomiting, constipation) Pancreatitis and biliary complications (rare)	Postoperative surgical complications (bleeding, blood clots, wound healing, leaks) Malabsorption issues and nutritional deficiencies
Type of therapy	Continuous	One time procedure

*Note*. GI = Gastrointestinal; GIP RA = Glucose-Dependent Insulinotropic Polypeptide Receptor Agonist; GLP1RA = Glucagon-Like Peptide-1 Receptor Agonists.

## 5. There Are Important Perioperative and Posttransplant Considerations in Obese Kidney Transplant Candidates and Recipients

For candidates on a GLP1RA at the time of transplantation, there are important perioperative considerations. GLP1RA have been associated with an increased risk of aspiration during anesthesia induction. However, the decision to discontinue a GLP1RA prior to surgery should be individualized based on aspiration risk versus therapeutic need, and the consequences of discontinuation (ie, hyperglycemia).^24^

From a posttransplant perspective, weight gain is common after kidney transplantation.^25^ Both GLP1RA and bariatric surgery have been shown to have important benefits in KTRs, although robust head-to-head studies in KTRs are currently lacking. Observational studies in KTRs treated with GLP1RA have demonstrated metabolic benefits of similar magnitude to nontransplant populations including reduction in body weight, HbA1c and proteinuria, with similar gastrointestinal side effects.^26^ Observational cohort studies suggest that GLP1RA use is associated with a lower risk of major adverse cardiovascular events, all cause-mortality and death-censored graft loss.^27,28^ Bariatric surgery in KTRs is also associated with significant weight loss, as well as improvements in obesity related comorbidities. In addition, bariatric surgery has been shown to improve both patient survival and graft survival.^29-31^ There appears to be no significant change in immunosuppressive drug levels with GLP1RA use in KTRs, unlike some forms of bariatric surgery.^26^

## Conclusion

Obesity in kidney transplant candidates is a common problem. Future studies should be aimed at understanding how to best integrate various weight loss strategies such lifestyle interventions, bariatric surgery and pharmacological therapies, which are rapidly evolving. Clinicians should focus on an individualized assessment of risk versus benefit, and a shared decision-making approach.
